# A regulatory loop containing miR-26a, GSK3β and C/EBPα regulates the osteogenesis of human adipose-derived mesenchymal stem cells

**DOI:** 10.1038/srep15280

**Published:** 2015-10-15

**Authors:** Zi Wang, Qing Xie, Zhang Yu, Huifang Zhou, Yazhuo Huang, Xiaoping Bi, Yefei Wang, Wodong Shi, Hao Sun, Ping Gu, Xianqun Fan

**Affiliations:** 1 Department of Ophthalmology, Ninth People’s Hospital, Shanghai Jiao Tong University School of Medicine, Shanghai, 200011, P.R. China

## Abstract

Elucidating the molecular mechanisms responsible for osteogenesis of human adipose-derived mesenchymal stem cells (hADSCs) will provide deeper insights into the regulatory mechanisms of this process and help develop more efficient methods for cell-based therapies. In this study, we analysed the role of miR-26a in the regulation of hADSC osteogenesis. The endogenous expression of miR-26a increased during the osteogenic differentiation. The overexpression of miR-26a promoted hADSC osteogenesis, whereas osteogenesis was repressed by miR-26a knockdown. Additionally, miR-26a directly targeted the 3′UTR of the GSK3β, suppressing the expression of GSK3β protein. Similar to the effect of overexpressing miR-26a, the knockdown of GSK3β promoted osteogenic differentiation, whereas GSK3β overexpression inhibited this process, suggesting that GSK3β acted as a negative regulator of hADSC osteogenesis. Furthermore, GSK3β influences Wnt signalling pathway by regulating β-catenin, and subsequently altered the expression of its downstream target C/EBPα. In turn, C/EBPα transcriptionally regulated the expression of miR-26a by physically binding to the CTDSPL promoter region. Taken together, our data identified a novel feedback regulatory circuitry composed of miR-26a, GSK3β and C/EBPα, the function of which might contribute to the regulation of hADSC osteogenesis. Our findings provided new insights into the function of miR-26a and the mechanisms underlying osteogenesis of hADSCs.

Mesenchymal stem cells (MSCs) have emerged as a promising tool for therapeutic applications in cell therapy and tissue engineering because of their ability to undergo tri-lineage differentiation into osteoblasts, chondrocytes and adipocytes^1^,^2^,^3^,^4^. MSCs isolated from various tissues (e.g., bone marrow, adipose tissue and umbilical cord blood^5^,^6^,^7^) have been used in potential treatments for various diseases and injuries including diabetes, graft-versus-host disease, myocardial infarction and spinal cord injury^8^,^9^,^10^,^11^. Adipose-derived mesenchymal stem cells (ADSCs) have great potential for use in bone regeneration because of their easy isolation, relative abundance, multipotency and rapid expansion^12^. Determining the molecular mechanisms responsible for osteogenesis of ADSCs will provide deeper insights into the regulatory patterns involved and will allow us to develop more efficient methods of cell-based therapies for treating bone defects.

MicroRNAs (miRNAs) are a class of endogenous, non-coding, single-strand RNAs, each composed of approximately 22–24 nucleotides. MiRNAs have been reported to incompletely complementarily bind to the 3′ untranslated region (3′UTR) of target mRNAs and interfere with the translation process, thus inhibiting protein synthesis^13^. Recent studies have revealed that miRNAs are involved in various biological processes including apoptosis, tumour and neuronal differentiation^14^,^15^,^16^,^17^. A cohort of miRNAs is differentially expressed in MSCs during the osteogenic differentiation process and has been reported to regulate the osteogenesis pathway through multiple mechanisms^18^,^19^,^20^. The up-regulation of miR-26a in MSCs during osteogenic differentiation has been reported by several research groups, indicating that miR-26a might participate in the regulation of osteogenesis^21^,^22^. However, the role of miR-26a in the regulation of the osteogenic differentiation of MSCs remains unclear as previous studies have described miR-26a as a negative regulator of osteogenesis^23^ but subsequent studies demonstrated that the overexpression of miR-26a promoted osteogenic differentiation^24^,^25^. Therefore, the role of miR-26a in the osteogenesis of hADSCs requires further investigation, and the regulatory mechanisms involved should also be explored.

Glycogen synthase kinase 3β (GSK3β) is an essential regulator of various biological processes that affect diverse molecular pathways including Wnt, PI3K/Akt and Hedgehog^26^,^27^,^28^,^29^. As a key component of the canonical Wnt signalling pathway, GSK3β along with a complex consisting of Axin1/2, APC and casein kinase 1 (CK1) constitutively degrade β-catenin through phosphorylation and the recruitment of the ubiquitin proteasome. Upon its dephosphorylation, β-catenin translocates into the cell nucleus and interacts with the T-cell factor/lymphoid enhancer factor-1 (TCF/LEF1) family of transcription factors, leading to the expression of target genes that are necessary for cell proliferation and differentiation^30^,^31^,^32^. The modulation of GSK3β through its phosphorylation or by chemical inhibitors has been shown to affect Wnt signalling pathway and to subsequently regulate the expression of various downstream target genes^33^,^34^,^35^,^36^. Recently, the regulation of GSK3β at the post-transcriptional level by miRNAs has also been demonstrated to impact the Wnt signalling pathway and diverse other biological processes^37^,^38^. MiR-26a has been demonstrated to be involved in the regulation of GSK3β and subsequently induces human airway smooth muscle hypertrophy and promotes apoptosis in hypoxic rat neonatal cardiomyocytes^39^,^40^. However, it remains unclear whether GSK3β is regulated by miR-26a in hADSCs and how miR-26a acts upon GSK3β, warranting further investigation. GSK3β has also been considered to participate in the regulation of osteogenic differentiation. Previous studies have demonstrated that the inhibition of GSK3β promotes osteogenic differentiation, but another study has revealed that the overexpression of GSK3β led to a marked increase in osteogenesis of murine ADSCs^41^,^42^,^43^. Thus, an investigation of the role of GSK3β in the regulation of the osteogenic differentiation of hADSCs would expand our knowledge of GSK3β’s diverse regulatory functions and could help explain the underlying mechanisms of miR-26a in the regulation of hADSC osteogenesis.

CCAAT-enhancer binding protein α (C/EBPα) has been demonstrated to be a major regulator in diverse physiological and pathological processes^44^,^45^, and it has been reported to regulate the expression levels of several miRNAs by physically binding to their promoter regions^46^,^47^. A previous study revealed that miR-26a could be transcriptionally activated by C/EBPα in human airway smooth muscle cells; specifically, a DNA fragment containing C/EBPα responsive elements within miR-26a promoter region could be immunoprecipitated by C/EBPα^40^. However, the transcriptional regulatory effects of C/EBPa on miR-26a in hADSCs remains unknown and requires further exploration to supply more precise information about the responsive elements and binding sites of C/EBPα within the miR-26a promoter region. C/EBPα has also been demonstrated to be one of various downstream target genes of the Wnt signalling pathway, and the activation of Wnt signalling has been shown to repress the expression of C/EBPα^48^,^49^. This raises the question as to whether GSK3β, a key component of the Wnt signalling pathway, also affects C/EBPα expression in hADSCs.

In this study, we analysed the effects of miR-26a on the osteogenesis of hADSCs by transducing lentiviral expression vectors that either promoted or repressed endogenous miR-26a. We identified GSK3β as one of the direct targets of miR-26a in hADSCs and further investigated the function of GSK3β in the regulation of hADSC osteogenic differentiation by performing gain- or loss-of-function analyses. In addition, we investigated the role of GSK3β in regulating β-catenin and revealed that C/EBPα is one of its downstream targets. C/EBPα was further demonstrated to transcriptionally regulate the expression of miR-26a. Taken together, our data suggested the existence of a feedback regulatory loop consisting of miR-26a, GSK3β and C/EBPα that regulates the osteogenesis of hADSCs.

## Methods and Materials

### Cell culture

Human adipose-derived mesenchymal stem cells (hADSCs) isolated from fat tissue were obtained from Cyagen Biosciences (Guangzhou, China) as previously described^50^. hADSCs were cultured in DMEM/F12 (Invitrogen, Carlsbad, CA, USA) supplemented with 10% FBS (Invitrogen) and 100 units/mL penicillin and streptomycin (Invitrogen). Passage 3 hADSCs were used for all experiments. hADSCs were cultivated in serum-free conditions for 24 h prior to stimulation with 10 mM lithium chloride (LiCl) from Amresco (Solon, OH, USA) for 24 h^51^. 293T cells were cultured in DMEM/F12 (Invitrogen) supplemented with 10% FBS (Invitrogen) and 100 units/mL each of penicillin and streptomycin (Invitrogen). All cells were incubated at 37 °C in a 5% CO_2_ humid atmosphere, and the cell medium was changed every 2–3 days.

### Reverse transcription and quantitative polymerase chain reaction (qPCR)

Total RNA was extracted from each sample using the RNeasy Mini Kit (Qiagen, Valencia, CA, USA), and first-strand complementary cDNA was synthesized using a PrimeScript™ RT reagent kit (Perfect Real Time, TaKaRa, Dalian, China). The resulting cDNAs were diluted 20-fold in nuclease-free water (Invitrogen) and were used as templates for qPCR. qPCR was carried out in a 20-μl solution containing 10 μl reaction mixture, 2 μl cDNA, and 300 nM of gene-specific primers designed using Primer 3 software (listed in Table 1). qPCR was conducted using a 7500 Real-Time PCR Detection System (Applied Biosystems, Irvine, CA, USA) with an activation at 95 °C for 10 min followed by 40 cycles of amplification (15 s at 95 °C and 1 min at 60 °C). The efficiency of the reaction was measured using primers with serial dilutions of cDNA (1:1, 1:5, 1:25, 1:125, 1:625 and 1:3,125)^52^. For miRNA qPCR, total RNA was extracted using RNeasy Mini Kit (Qiagen), and 1 μg of total RNA was reverse-transcribed using stem-loop primers from a BioTNT miRNA qPCR Detection Primer Set (BioTNT Biotechnologies, China). Each sample was tested in triplicate. The relative gene expression levels of mRNA and miRNA were analysed using the Pfaffl method^53^ in which GADPH and U6B were used as endogenous normalization controls.

### Lentiviral construction and transduction

The lentiviral expression vector expressing hsa-miR-26a was termed miR-26a. Total RNA was first extracted from hADSCs, and cDNA was generated by RT-PCR. The target amplicon was generated using the primer listed in Table 2 and was cloned into a pLenti-Ubi-EGFP vector (Genechem Technology, China). The lentiviral expression vector expressing the reverse complementary sequence of hsa-miR-26a was termed miR-26a inhibitor. The oligonucleotide containing the stem-loop structure was synthesized as shown in Table 3 and cloned into a pLenti-hU6-EGFP vector (Genechem). 293T cells were transfected with the lentiviral expression vectors and packing vectors including Gag-Pol and VSV-G (all from Genechem). Forty-eight hours after transfection, supernatants containing virus were collected and then filtered and concentrated by a Centricon Plus-20 filter device (Millipore Corporation, Billerica, MA, USA). For lentiviral transduction, the cell medium was first changed into Opti-MEM (Invitrogen) with 5 μg/mL of polybrene (GeneChem), and an optimal volume of concentrated viral supernatants was added.

### Plasmid construction

The cDNA of C/EBPα (NM_004364) was generated by RT-PCR using the primers listed in Table 2, and the cDNA of GSK3β (NM_001146156) was purchased from GeneChem. The two cDNAs were individually cloned into a pcDNA3.1 vector (Invitrogen) and termed p-C/EBPα and p-GSK3β, respectively; empty pcDNA3.1 vector was termed p-NC and used as a control. To generate the luciferase reporter vector, a 199-bp fragment of GSK3β (NM_001146156) 3′UTR containing the predicted miR-26a binding site (position 4636–4643) and its mutant sequence were synthesized by Genechem and cloned into pGL3-control vector (Promega (Beijing) Biotech Co., Ltd, China); the constructed sequences are listed in Table 3. The full-length GSK3β 3′UTR containing either the predicted binding site (position 4636–4643) or its mutant sequence was synthesized by GeneChem and cloned into a pGL3-control vector (Promega); these constructs were termed GSK3β 3′UTR-full-wt and GSK3β 3′UTR-mut, respectively. The two 1000-bp fragments within the CTDSPL promoter region (-2000/-1001 and -1000/-1 from ATG) were synthesized by GeneChem and inserted upstream into a pGL3-basic vector (Promega); the constructs were termed pGL3-Promoter1 and 2, respectively. Three fragments with some sequences deleted were synthesized and cloned upstream into a pGL3-basic vector (Promega); these constructs were termed pGL3-ΔA (-1580/-1464, 117-bp deletion), pGL3-ΔB (-1422/-1301, 122-bp deletion) and pGL3-ΔC (-1220/-1101, 120-bp deletion). The two 1000-bp (-2000/-1001) sequences containing either the wild type (-1530/-1526, GCAAG) or mutant (-1530/-1526, ATGGA) binding sites of C/EBPα were synthesized and inserted upstream into a pGL3-basic vector (Promega); these constructs were termed pGL3-wild type and pGL3-mutant, respectively. A pRL-TK vector expressing renilla luciferase was obtained from Promega and used as an endogenous normalizer.

### Plasmids and siRNA transfection

hADSCs were seeded in 6-well plates before transfection. The transfection was conducted in Opti-MEM (Invitrogen), and the transfection mix was composed of 3 μg of each plasmid and an optimal volume of Lipofectamine 2000 Reagent (Invitrogen). After 8 h of transfection at 37 °C in a humidified environment containing 5% CO_2,_ the medium was changed, and the cells were incubated for another 48 h. Three pairs of small interfering RNAs (siRNAs) were designed and synthesized by Biomics (Biomics Biotechnologies, Shanghai, China) to specifically degrade the mRNAs of β-catenin (NM_001904) and GSK3β (NM_001146156). These siRNAs are listed in Table 3 and termed si-β-catenin-1, 2 and 3 and si-GSK3β-1, 2 and 3, respectively. Negative control siRNA was used and termed si-NC. The siRNAs were transfected into hADSCs using the same method at a final concentration of 50 nM^50^.

### Western blot analyses

Western blot analyses were performed using a standard protocol as previously described^54^. Confluent hADSCs were lysed with RIPA lysis buffer (Beyotime Institute of Biotechnology, China) supplemented with 1 nM of PMSF (Invitrogen), after which the collected protein contents were measured using a BCA protein assay kit (Thermo Fisher Scientific Inc., Waltham, MA). Proteins were separated by 10% SDS-PAGE electrophoresis and electro-blotted onto PVDF membranes (Millipore). The membranes were then incubated with optimal concentrations of the following primary antibodies: anti-Runx2 (1:1500, Abcam, Cambridge, MA, USA), anti-Ocn (1:1000, Abcam), anti-BSP (1:1000, Abcam), anti-GSK3β (1:1000, Abcam), anti-β-catenin (1:2000, Abcam), anti-C/EBPα (1:1000, Abcam) and anti-β-actin (1:3000, Abcam). Immunoreactive bands were detected using anti-rabbit (1:5000) or anti-mouse (1:5000) fluorescein-conjugated secondary antibodies (Abcam) and visualized by Odyssey V3.0 image scanning. All of the procedures were performed three times.

### Quantitative ALP and calcium measurements and ALP and ARS staining

hADSCs were treated with osteogenic induction medium (StemPro® Osteogenesis Differentiation Kit, Invitrogen) for 14 days following lentiviral transduction or plasmid/siRNA transfection. For quantitative alkaline phosphatase (ALP) measurements, cells were first lysed using RIPA lysis buffer (Beyotime), and the cell supernatant was collected into a 96-well plate prior to the addition of substrates and p-nitrophenol from an Alkaline Phosphatase Assay Kit (Beyotime). After 15-minutes incubation at 37 °C, ALP activity was measured at a wavelength of 405 nm. Calcium content measurements were conducted using a Calcium Colorimetric Assay Kit (Biovision, CA, USA) according to the manufacturer’s instructions. Briefly, the cells and extracellular matrices were washed and diluted in the buffer solution, and the active solution was added into each well. After 15-minutes incubation, the measurement of calcium was conducted at a wavelength of 575 nm. ALP and alizarin red s (ARS) staining were performed as previously described^54^. Cells were washed and fixed in 4% polyoxymethylene for 10 min, and ALP staining was then performed using an Alkaline Phosphatase Color Development Kit (Beyotime) according to the manufacturer’s instructions by incubating the cells for 30 min at 37 °C. For ARS staining, hADSCs were first washed and fixed in cold 95% (v/v) ethanol for 30 min, and the fixed cells were subsequently incubated with staining solution (Sigma-Aldrich, St. Louis, MO, USA) at 37 °C for 30 min.

### Dual luciferase reporter assay

A total of 0.4 μg of pGL3-control (Promega) plasmid containing either the wild type or the mutant miR-26a binding site, 0.3 μg of pRL-TK (Promega) plasmid containing the renilla luciferase reporter gene, and 0.3 μg of miR-26a expressing vector were co-transfected into 293T cells using the Lipofectamine 2000 Reagent (Invitrogen). A total of 0.2 μg of pGL3-basic (Promega) plasmid containing the CTDSPL promoter region, 0.6 μg of C/EBPα expressing vector and 0.05 μg of pRL-TK were transfected using the same method. For the Wnt signalling pathway, 0.3 μg of pGL4-luc2P/TCF-LEF/Hygro vector containing TCF/LEF responsive elements were purchased directly from Promega and were co-transfected into hADSCs with either 0.6 μg of p-GSK3β or 50 nM of si-GSK3β. For this assay, 0.3 μg of pRL-TK (Promega) was used as a normalizer. Cells were harvested 48 h after transfection and assayed for firefly and renilla luciferase activity using the Dual-Glo™ Luciferase Assay System (Promega). The firefly luciferase activity was normalized to the renilla luciferase activity.

### Cellular immunofluorescence and CLSM imaging

Cellular immunofluorescence was conducted as previously described^55^. hADSCs were first fixed in 4% paraformaldehyde (Sigma) and then permeabilized with 1% Triton X-100 (Invitrogen). The cells were incubated with an optimal concentration of rabbit anti-β-catenin antibody (1:500, Abcam), anti-Ostrix antibody (1:500, Abcam), anti-SATB2 antibody (1:500, Abcam) and anti-Runx2 antibody (1:500, Abcam) overnight at 4 °C followed by an incubation with anti-rabbit Alexa Fluor 546 secondary antibody (1:2000, Invitrogen), and the cells were subsequently rinsed five times with PBS. Nuclei were stained with Hoechst (Invitrogen) prior to imaging on a Leica TCS SP8 microscope (Leica Microsystems, Germany). Images were constructed using Leica LAS AF software (Leica).

### Chromatin immunoprecipitation (ChIP)

ChIP was performed as previously described^56^. When cellular confluence reached 80%, hADSCs were cross-linked with 1% formaldehyde (Sigma) at 37 °C for 15 min. The cells were then lysed, and DNA-protein complexes were immunoprecipitated. Next, the formaldehyde-cross-linked DNA was reverse cross-linked using a ChIP Assay Kit (Millipore) according to the manufacturer’s protocol. DNA-chromatin complexes were immunoprecipitated with anti-C/EBPα (1:300, Abcam) or mouse IgG (Millipore) as an internal control. The primers used for analysing the precipitated DNA are listed in Table 2.

### Bioinformatics predictions

To predict the target genes of miR-26a during the osteoblast differentiation of hADSCs, we selected scientifically sanctioned miRNA target prediction databases: TargetScan (www.targetscan.org) and miRanda (www.miranda.org); and for the prediction of C/EBPα binding sites, Patch 1.0 (www.gene-regulation.com) was used.

### Statistical analyses

The results represent the average of three experiments, and the data are presented as the mean ± SD. Each experiment was performed at least three times unless otherwise specified. Statistical significance was determined using the unpaired Student’s t-test, and a value of *P < 0.05 was considered to be statistically significant.

## Results

### miR-26a promotes the osteogenesis of hADSCs

**Figure 1:**
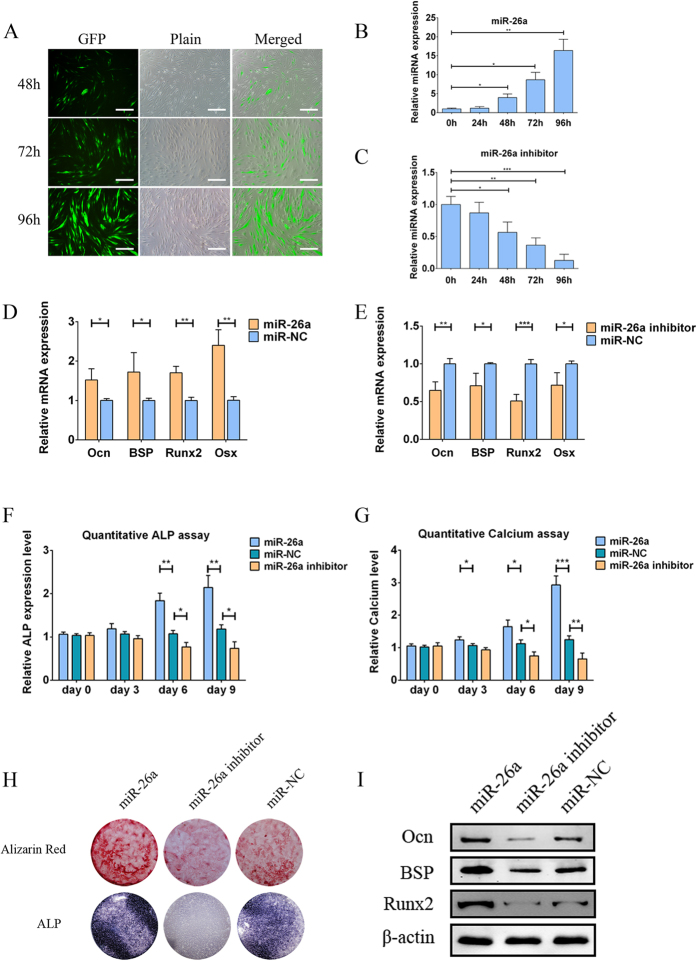
miR-26a promoted the osteogenesis of hADSCs. (**A**) The GFP expression levels hADSCs after lentiviral transduction were imaged by a fluorescence microscope, showing that the ratio of GFP-positive hADSCs was greater than 70% at 96 hours. Scale bars: 500 μm. Intracellular miR-26a levels were greatly increased by the lentiviral transduction of miR-26a (**B**) and were remarkably decreased by a miR-26a inhibitor in a time-dependent manner (**C**). (**D**) mRNA expression levels of osteogenic-specific genes such as Ocn, BSP, Runx2 and Osx were increased by miR-26a transduction. (**E**) The silencing of intracellular miR-26a by transducing miR-26a inhibitor repressed the mRNA levels of the osteogenic-specific genes. Data shown represented the average of three independent experiments and were all normalized to GADPH. (**F**) A quantitative ALP assay indicated that ALP activity was increased by miR-26a, but decreased by the miR-26a inhibitor compared to the miR-NC group. (**G**) A quantitative calcium assay indicated that calcium content was increased by miR-26a but was reduced by the miR-26a inhibitor compared to miR-NC. Data from each time point were normalized to the miR-NC group at day 0. (**H**) ALP and ARS staining revealed that the exogenous overexpression of miR-26a increased intracellular ALP levels and mineralized the extracellular matrix, whereas the miR-26a inhibitor reduced these levels. (**I**) Western blot showed that the protein levels of the osteogenic-specific genes such as Ocn, BSP and Runx2 were elevated by miR-26a but repressed by the miR-26a inhibitor. These data are representative of at least three independent experiments; β-actin was used as a normalizer. Data are averages of three independent experiments. *P < 0.05, **P < 0.01, ***P < 0.001.

To investigate the expression pattern of endogenous miR-26a during the osteogenic differentiation process, hADSCs were treated with either osteogenic medium or normal medium, and the expression levels of miR-26a were detected at each time point by qPCR. As shown in Supplementary Fig. S1A online, miR-26a expression was gradually up-regulated in hADSCs cultured in osteogenic medium compared with those cultured in normal medium. To elucidate the role of miR-26a in regulating the osteogenic differentiation of hADSCs, a total of three lentiviral expression systems were constructed: a lentiviral vector overexpressing miR-26a (miR-26a); a lentiviral vector expressing the complementary sequence of mature miR-26a (miR-26a inhibitor); and a lentiviral vector without any insertions of expression sequences (miR-NC), which was used as control. Then, hADSCs were transduced with the lentiviral expression systems, and GFP expression was imaged by a fluorescence microscope to detect the transduction efficiency. As shown in Fig. 1A, the ratio of GFP-positive hADSCs increased in a time dependent manner, reaching 71.7 ± 5.63% at 96 hours. qPCR analyses showed that intracellular miR-26a was remarkably elevated by the transduction of miR-26a, whereas its expression was reduced to less than 30% by the transduction of miR-26a inhibitor (Fig. 1B,C). Next, we performed qPCR 7 days after the transduction, which revealed that the overexpression of miR-26a resulted in an increase in the mRNA expression levels of several osteogenic marker genes, such as Ocn, BSP, Runx2 and Osx, to at least 1.5-fold compared to miR-NC (Fig. 1D). In contrast, the knockdown of miR-26a by the transduction of miR-26a inhibitor decreased those same mRNA levels by as much as 50% compared to miR-NC (Fig. 1E). Important osteogenic marker alkaline phosphatase (ALP) activity was measured by a quantitative ALP assay and showed that the overexpression of miR-26a promoted ALP activity; ALP activity was decreased by the knockdown of miR-26a. Data from each time point was compared to miR-NC at the same time point (Fig. 1F). In addition, we performed a quantitative calcium assay to determine the contents of the mineralized extracellular matrix (ECM), and this assay showed a similar pattern to the ALP assay (Fig. 1G). ALP and alizarin red s (ARS) staining were also performed 14 days after transduction to generally observe miR-26a’s effects on ALP activity and mineralization of ECM. As shown in Fig. 1H, miR-26a-transduced hADSCs exhibited both increased ALP activity and more mineralized ECM, whereas the miR-26a inhibitor led to reduction of both. Furthermore, western blot analyses was performed 7 days after transduction to detect the protein levels of genes related to osteogenesis, and the protein levels of Ocn, BSP and Runx2 were increased in miR-26a-transduced hADSCs while decreased in miR-26a inhibitor-transduced hADSCs (Fig. 1I). Overall, these data suggested that the overexpression of miR-26a promoted the osteogenic differentiation of hADSCs whereas the knockdown of miR-26a repressed it, indicating that miR-26a was a positive regulator for the osteogenesis of hADSCs.

### GSK3β is a direct target of miR-26a

**Figure 2:**
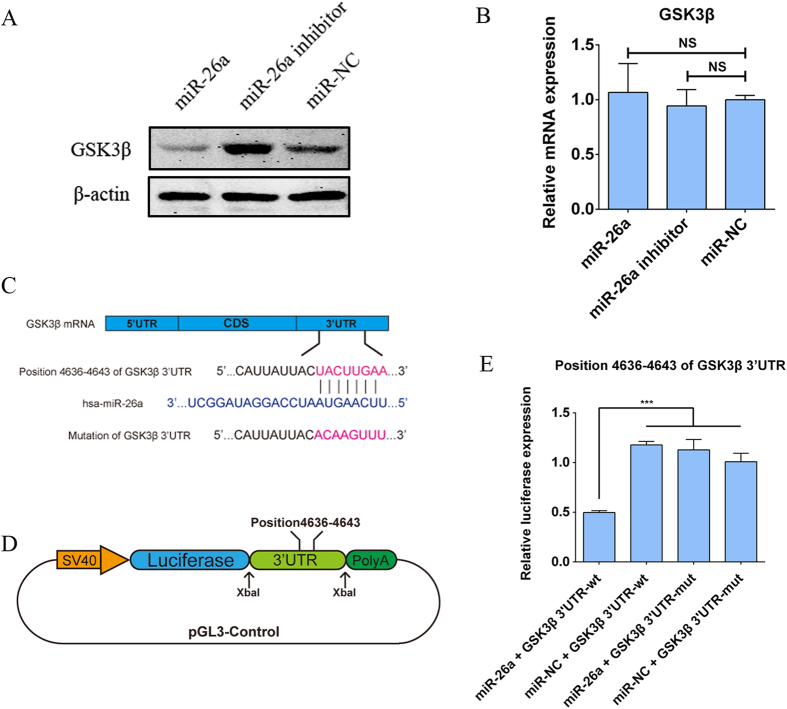
GSK3β is a direct target of miR-26a. (**A**) Western blot analyses indicated that the GSK3β expression level was inhibited by miR-26a but was promoted by miR-26a inhibitor. (**B**) qPCR showed that neither the exogenous miR-26a nor the miR-26a inhibitor had significant impacts on GSK3β mRNA levels. qPCR data are the averages of three independent experiments and were all normalized to GADPH. NS stands for no significance observed. (**C**) Position 4636–4643 of the 3′UTR of GSK3β mRNA and its mutated sequence are presented in a schematic diagram. (**D**) Schematic diagram of the luciferase reporter system that was constructed, which contained either wild type or mutant binding sites. (**E**) The dual luciferase reporter assay indicated that the co-transfection of miR-26a and the wild-type binding site (GSK3β 3′UTR-wt) dramatically reduced luciferase activity, whereas miR-26a had no effects on the mutated binding region (GSK3β 3′UTR-mut). All data are averages from three independent experiments. The firefly luciferase activity data were normalized to renilla luciferase activity. ***P < 0.001.

To test our hypothesis of whether GSK3β is a direct target of miR-26a, hADSCs were transduced with miR-26a, miR-26a inhibitor or miR-NC, and the results of western blot showed that the protein levels of GSK3β was repressed by miR-26a but promoted by miR-26a inhibitor compared to miR-NC (Fig. 2A). Our qPCR analyses detected no significant changes in the levels of GSK3β mRNA (Fig. 2B), suggesting that miR-26a regulated the expression of GSK3β at the post-transcriptional level. To further validate whether miR-26a directly interacted with GSK3β 3′UTR and subsequently interfered with the translation process, we searched miRanda and TargetScan, where a more negative score indicates a greater likelihood of being a direct binding site of miR-26a. We found out several miR-26a putative binding sites whose score ranges from −0.0022 to −0.7538 (miRanda) and −0.120 to −0.247 (TargetScan). Among these binding sites, position 4636–4643 had the most negative score (−0.7538 in miRanda, and −0.247 in TargetScan), indicating that miR-26a could directly bind to this site. To test this binding site, a luciferase reporter system was constructed. A 199-bp fragment of the GSK3β 3′UTR containing either the wild type or mutant sequences of position 4636–4643 was cloned downstream of the firefly luciferase coding sequence in the pGL3-control vector (Fig. 2D); these constructs were termed GSK3β 3′UTR-wt and GSK3β 3′UTR-mut, respectively. Then, miR-26a-overexpressing plasmid (miR-26a) or empty plasmid (miR-NC) was individually co-transfected with GSK3β 3′UTR-wt or GSK3β 3′UTR-mut, and the renilla luciferase plasmid (pRL-TK) was used to normalise the expression. Luciferase assays showed that the co-transfection of miR-26a and GSK3β 3′UTR-wt dramatically decreased the luciferase activity compared with the other groups (Fig. 2E), indicating that position 4636–4643 of the 3′UTR of GSK3β was a direct target of miR-26a. To obtain a full picture of the post-transcriptionally repressive effects of miR-26a on GSK3β and to exclude the possibility that secondary structures of the full-length 3′-UTR were hampering the recognition of the particular binding site, we also synthesized the full-length 3′UTR of GSK3β carrying either the wild-type or mutant sequences of position 4636–4643. These sequences were then inserted into the pGL3-control vector, and the constructs were termed GSK3β 3′UTR-full-wt and GSK3β 3′UTR-full-mut, respectively. As shown in Supplementary Fig. S1B online, the co-transfection of miR-26a and GSK3β 3′UTR-full-wt significantly repressed the luciferase expression, whereas the co-transfection of miR-26a and GSK3β 3′UTR-full-mut showed little effect on luciferase expression. Taken together, our data showed that miR-26a suppressed the protein levels of GSK3β, whereas the knockdown of miR-26a increased GSK3β protein expression. Furthermore, miR-26a repressed the translation of GSK3β by directly binding to position 4636–4643 of the GSK3β 3′UTR. These findings suggested that GSK3β was a direct target of miR-26a.

### GSK3β is a negative regulator of hADSC osteogenesis

**Figure 3:**
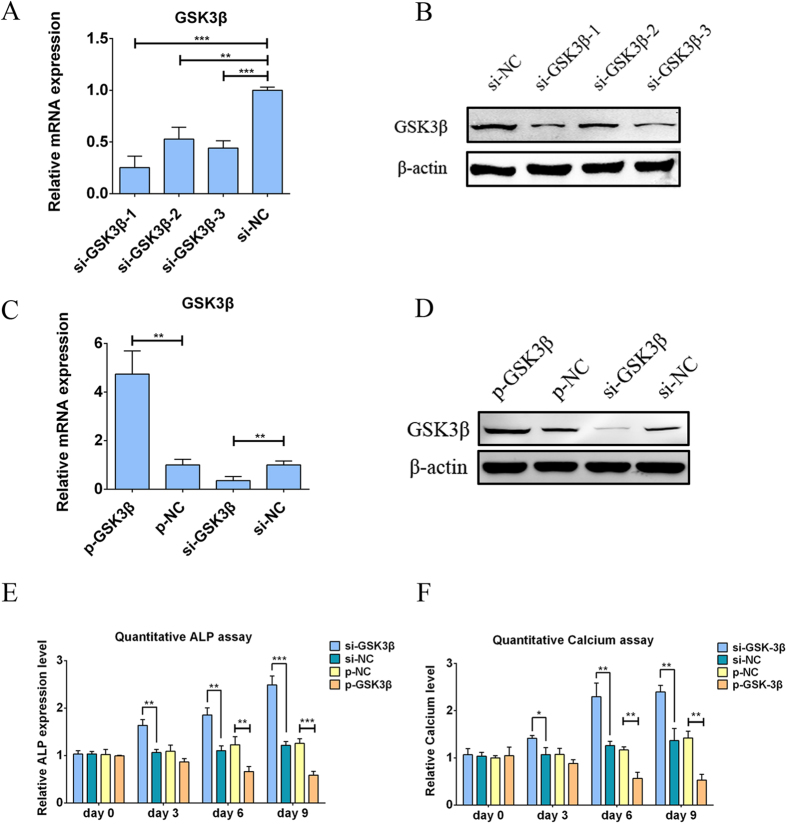
GSK3β represses the osteogenesis of hADSCs. (**A**) qPCR detection showed that the transfection of si-GSK3β resulted in a sharp decrease in the mRNA levels of GSK3β. (**B**) Western blot indicated that the transfection of si-GSK3β significantly reduced GSK3β protein levels . (**C**) qPCR showed that the GSK3β overexpression plasmid resulted in a marked increase in the mRNA levels of GSK3β, whereas the transfection of si-GSK3β reduced the mRNA levels of GSK3β. (**D**) Western blot showed similar results, namely, that GSK3β protein levels were elevated by p-GSK3β and that they were decreased by si-GSK3β. (**E**) A quantitative ALP assay showed that p-GSK3β decreased ALP activity whereas si-GSK3β increased it. Data from each time point were normalized to p-NC and si-NC, respectively, at Day 0. (**F**) A quantitative calcium assay showed that the overexpression of GSK3β reduced the calcium content but that si-GSK3β increased it. Data from each time point were normalized to p-NC and si-NC, respectively, at Day 0. Data are averages of three independent experiments. All data of qPCR were normalized to GADPH, p-NC and si-NC were used as the negative control of p-GSK3β and si-GSK3β, respectively. *P < 0.05, **P < 0.01, ***P < 0.001.

**Figure 4:**
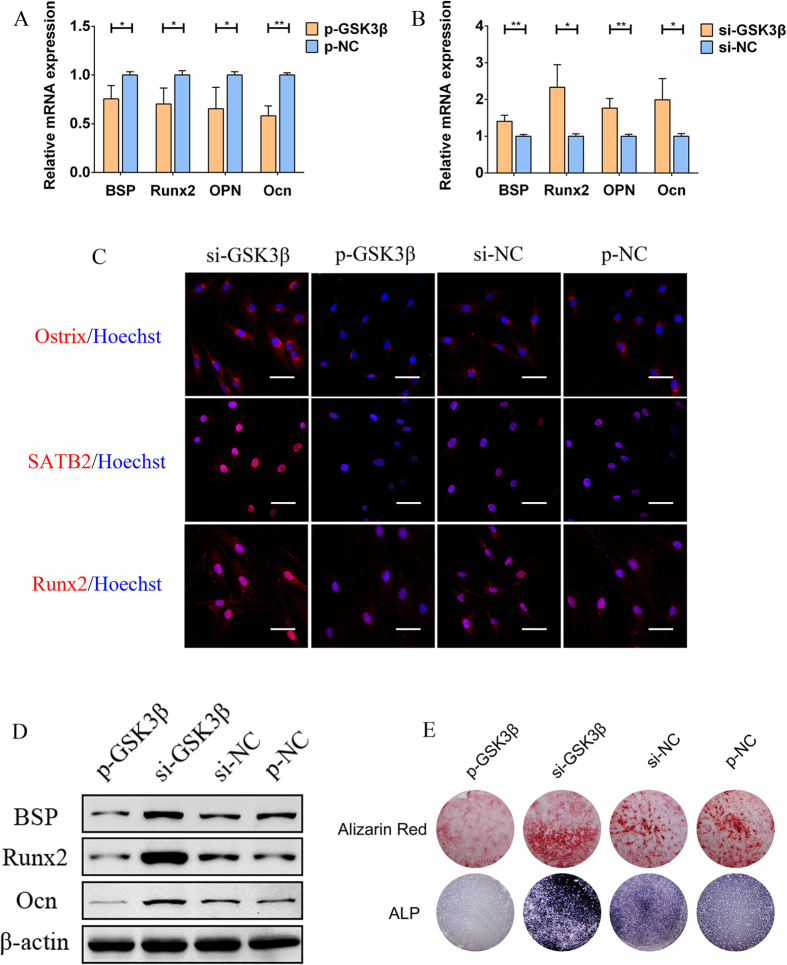
GSK3β is a negative regulator of the osteogenesis of hADSCs. (**A**) qPCR detection revealed that the overexpression of GSK3β reduced the mRNA levels of osteogenic-related genes such as BSP, Runx2, OPN and Ocn. (**B**) The siRNA-mediated knockdown of GSK3β promoted the mRNA levels of BSP, Runx2, OPN and Ocn. (**C**) Cellular immunofluorescence imaged by confocal laser scanning microscope (CLSM) showed that the expression levels of Ostrix, SATB2 and Runx2 were repressed by the overexpression of GSK3β but were promoted by the knockdown of GSK3β. Scale bars: 100 μm. (**D**) Western blot analyses showed that p-GSK3β repressed and si-GSK3β enhanced the protein levels of osteogenesis-specific genes such as Ocn, BSP and OPN. (**E**) ALP and ARS staining indicated that the overexpression of GSK3β reduced the presence of intracellular ALP and of the mineralized extracellular matrix in hADSCs, whereas both were increased by the knockdown of GSK3β. All of the data are averages of three independent experiments. All qPCR data were normalized to GADPH; p-NC and si-NC were set to be the negative controls of p-GSK3β and si-GSK3β, respectively. *P < 0.05, **P < 0.01.

To investigate the effects of GSK3β on the osteogenesis of hADSCs, we constructed a GSK3β overexpression plasmid, termed p-GSK3β, and designed three pairs of siRNA to degrade the mRNA level of GSK3β, called si-GSK3β-1, 2 and 3; empty plasmid (p-NC) and negative control siRNA (si-NC) were used as controls. To test the knockdown efficiency, three pairs of si-GSK3β were individually transfected into hADSCs, qPCR and western blot were performed. Both the mRNA (Fig. 3A) and protein (Fig. 3B) levels of GSK3β were significantly decreased by the transfection of the three pairs of si-GSK3β, among which si-GSK3β-3 showed the strongest inhibition and was selected for subsequent GSK3β knockdown experiments. To validate the effects of p-GSK3β and si-GSK3β on the expression of GSK3β in hADSCs, p-GSK3β and si-GSK3β were individually transfected into hADSCs, and the mRNA and protein expression levels of GSK3β were examined by qPCR and western blot, respectively. As shown in Fig. 3C,D, both the mRNA and protein levels were dramatically elevated by p-GSK3β, whereas these levels were repressed by si-GSK3β, indicating that p-GSK3β and si-GSK3β could be used to modulate intracellular GSK3β levels. Next, p-GSK3β and the si-GSK3β were individually transfected into hADSCs to investigate the role of GSK3β in the regulation of hADSC osteogenesis. Important osteogenic marker ALP activity was determined by a quantitative ALP assay, which showed that intracellular ALP activity (Fig. 3E) was greatly promoted by si-GSK3β but was significantly repressed by p-GSK3β; Data from p-GSK3β or si-GSK3β at each time point was individually compared to p-NC or si-NC at the same time point. In addition, quantitative calcium assay was performed to evaluate the mineralization of ECM, which showed a similar pattern as the quantitative ALP assay (Fig. 3F). Furthermore, qPCR at day 7 showed that the mRNA levels of osteogenic marker genes such as BSP, Runx2, OPN and Ocn were repressed by the transfection of p-GSK3β (Fig. 4A) but were elevated by si-GSK3β (Fig. 4B) compared to controls. To evaluate the effects of GSK3β on osteogenic-specific transcription factors in hADSCs, cellular immunofluorescence was conducted 7 days following transfection. As shown in Fig. 4C, the expression levels of Ostrix, SATB2 and Runx2 were repressed by the transfection of p-GSK3β but were elevated in si-GSK3β-transfected hADSCs compared to controls. Seven days after the transfection, the protein levels of osteogenic marker genes such as Ocn, BSP and OPN were also decreased by p-GSK3β while being promoted by si-GSK3β compared to controls (Fig. 4D). ALP and ARS staining (Fig. 4E) was conducted 14 days after the transfection to generally observe the ALP activity and mineralization of ECM, which showed that p-GSK3β decreased both intracellular ALP activity and mineralized ECM in hADSCs; both were increased following si-GSK3β transfection. Collectively, our data suggested that the overexpression of GSK3β enhanced the osteogenic differentiation of hADSCs whereas the knockdown of GSK3β repressed this differentiation, indicating that GSK3β acts as a negative regulator of hADSC osteogenesis.

### GSK3β regulates β-catenin and its downstream target C/EBPα.

**Figure 5:**
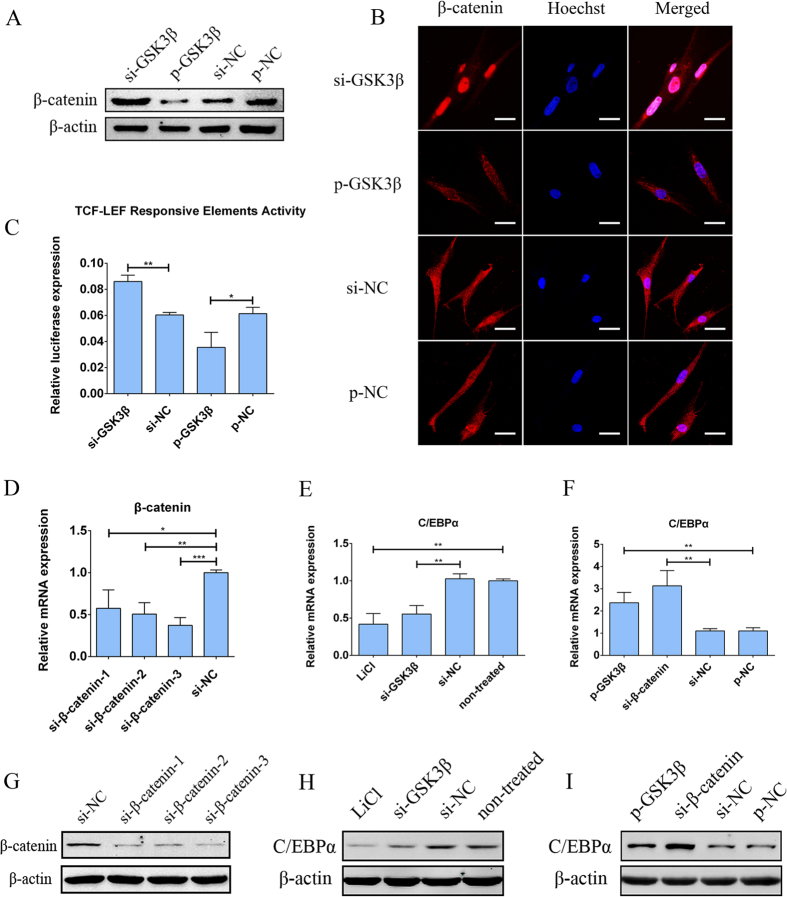
GSK3β regulates β-catenin and its downstream target C/EBPα. (**A**) Western blot showed that β-catenin was increased by si-GSK3β and repressed by p-GSK3β. (**B**) Our cellular immunofluorescence assay indicated that the intracellular content of β-catenin increased and shifted from the cytoplasm to the nucleus when si-GSK3β was transfected but that the overexpression of GSK3β reduced β-catenin levels. Scale bars: 50 μm. (**C**) A luciferase reporter assay with TCF/LEF responsive elements showed that the β-catenin-TCF/LEF-driven luciferase expression level was elevated by si-GSK3β but decreased by p-GSK3β. All data are averages from three independent experiments. p-NC and si-NC were used as the negative controls for p-GSK3β and si-GSK3β, respectively. The firefly luciferase activity data were normalized to renilla luciferase. The transfection of si-β-catenin led to a sharp reduction in mRNA (**D**) and protein (**G**) levels of β-catenin. qPCR indicated that the mRNA levels of C/EBPα decreased when hADSCs were transfected with si-GSK3β or treated with 10 mM of LiCl, compared with si-NC or non-treated hADSCs, respectively, (**E**), but that C/EBPα levels were increased following the transfection of si-β-catenin or p-GSK3β compared with si-NC or p-NC (**F**). Western blot also showed that C/EBPα protein levels increased in LiCl-treated or si-GSK3β-transfected hADSCs (**H**) whereas they decreased in si-β-catenin or p-GSK3β-transfected hADSCs (**I**). All data are averages from three independent experiments, and data of qPCR were all normalized to GADPH. *P < 0.05, **P < 0.01, ***P < 0.001.

It has been reported that the activation of Wnt signaling pathway leads to intracellular accumulation of β-catenin, and accumulated β-catenin subsequently translocates into the cell nucleus and interacts with the T-cell factor/lymphoid enhancer factor-1 (TCF/LEF1) family of transcription factors, leading to the expression of target genes^29^,^30^. C/EBPα has been shown to be one of the downstream target genes of the Wnt pathway^48^,^49^, and we tested whether GSK3β, one of the key components of the Wnt pathway, regulates C/EBPα expression in hADSCs. First to investigate the role of GSK3β in regulating the Wnt pathway, hADSCs were individually transfected with si-GSK3β and p-GSK3β; empty plasmid (p-NC) and negative control siRNA (si-NC) were used as controls. Western blot analyses showed that intracellular β-catenin levels were elevated following the siRNA-mediated knockdown of GSK3β, whereas intracellular β-catenin levels were repressed by the overexpression of GSK3β compared to controls (Fig. 5A). Cellular immunofluorescence showed that si-GSK3β elevated intracellular β-catenin levels and promoted its nuclear aggregation, whereas p-GSK3β reduced β-catenin levels and prevented β-catenin from translocating into the nucleus (Fig. 5B). Next, a luciferase reporter vector containing TCF/LEF responsive elements was co-transfected with si-GSK3β or p-GSK3β into hADSCs, and a luciferase assay showed that the activity of TCF/LEF responsive elements was significantly increased by the knockdown of GSK3β; the activity of TCF/LEF elements was decreased by the overexpression of GSK3β (Fig. 5C). These findings suggested that GSK3β regulated both the intracellular level and localization of β-catenin in hADSCs, consequently affecting the Wnt signalling pathway.

Based on our results that GSK3β regulates β-catenin and Wnt signaling pathway, we further investigated whether GSK3β could regulate C/EBPα expression in hADSCs. According to previous studies, LiCl is a strong activator of the Wnt pathway, and the knockdown of β-catenin using siRNA significantly represses the Wnt pathway^51^, thus they were employed in our study as positive control. First, to select the most effective siRNA that could knockdown β-catenin, we designed three pairs of siRNA termed si-β-catenin-1, 2 and 3; negative control siRNA (si-NC) was used as a control. Then, three pairs of si-β-catenin were individually transfected into hADSCs; qPCR (Fig. 5D) and western blot (Fig. 5G) analyses showed that both the mRNA and protein levels of β-catenin were significantly decreased by the transfection of three pairs of si-β-catenin. Among these, si-β-catenin-1 was selected for the following experiments to knockdown β-catenin. To investigate the influence of GSK3β on C/EBPα, P-GSK3β and si-GSK3β were individually transfected into hADSCs. qPCR (Fig. 5E) and western blot (Fig. 5H) analyses showed that the treatment with LiCl or the transfection of si-GSK3β significantly decreased C/EBPα expression compared to non-treated hADSCs and si-NC; the knockdown of β-catenin by si-β-catenin or the transfection of p-GSK3β greatly promoted C/EBPα expression compared with si-NC and p-NC, respectively (Fig. 5F,I). These findings suggest that C/EBPα is one of the downstream targets of the Wnt signalling pathway and is negatively regulated by GSK3β. Above all, our data demonstrated that GSK3β has significant impacts on the Wnt signalling pathway by affecting β-catenin and consequently regulates the expression level of its downstream target C/EBPα.

### C/EBPα regulates miR-26a by directly binding to its promoter region

**Figure 6:**
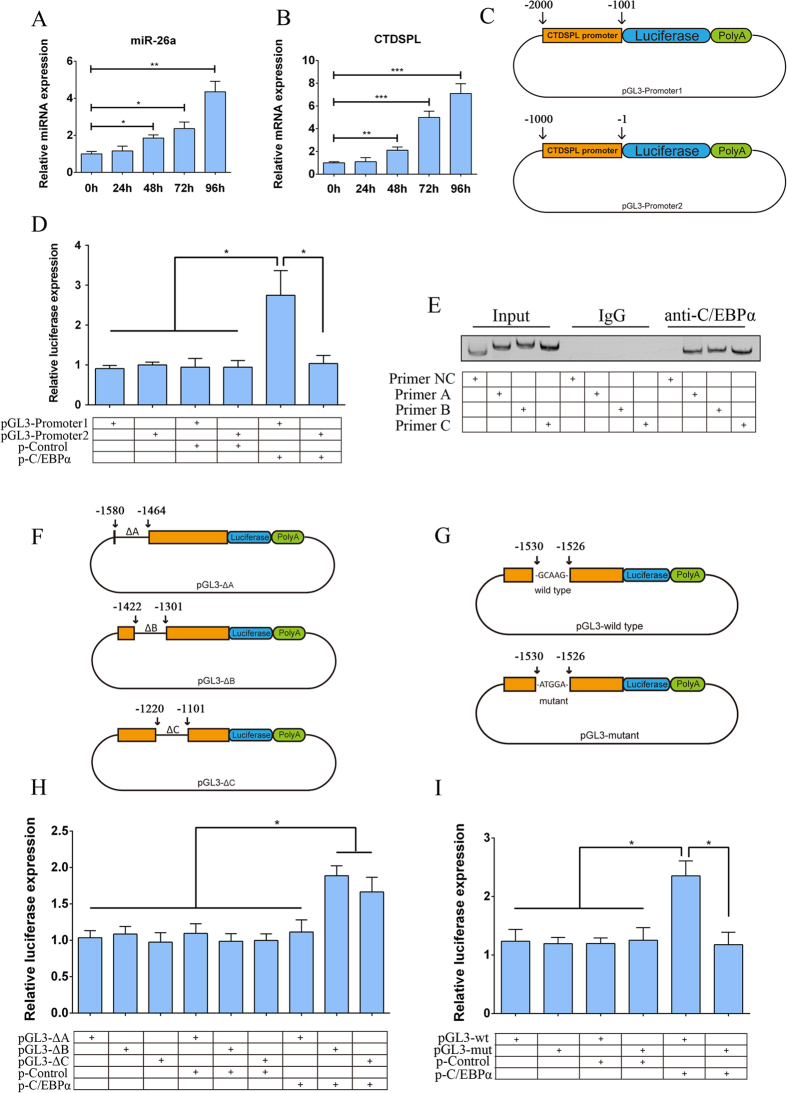
C/EBPα regulates miR-26a by directly binding to its promoter region. (**A**) qPCR detection showed that the exogenous transfection of C/EBPα elevated the expression of miR-26a; data were normalized to U6B. (**B**) The transfection of C/EBPα promoted the expression of CTDSPL; data were normalized to GADPH. (**C**) Schematic diagram of the two luciferase reporter systems constructed to assess promoter activity. (**D**) Our luciferase activity assay indicated that the co-transfection of the pGL3-Promoter1 and p-C/EBPα increased luciferase expression levels. (**E**) ChIP assays showed that three fragments within the promoter region (-2000/-1001 and -1000/-1 from the ATG of CTDSPL) could be immunoprecipitated by C/EBPα and amplified using Primer A (-1580/-1464, 117 bp), B (-1422/-1301, 122 bp) and C (-1220/1101, 120 bp), Primer NC (-1786/-1683, 104 bp) was set as a negative control. ChIP assays were performed under the following conditions: using no antibody (input), using C/EBPα antibody (C/EBPα) and using the control IgG antibody (IgG). (**F**) The schematic diagram represents the three luciferase reporter systems with some sequence deletion constructed to investigate the transcriptional activity of fragments that were immunoprecipitated by C/EBPα in ChIP assays. (**G**) Luciferase assay showed that co-transfection of pGL3-ΔA and p-C/EBPα no longer promoted luciferase expression as pGL3-ΔB and pGL3-ΔC did. (**H**) Schematic diagram represents the luciferase reporter systems containing either the wild type (-1530/-1526, GCAAG) or mutant (-1530/-1526, ATGGA) binding site of C/EBPα. (**I**) Luciferase assay showed that co-transfection of pGL3-mutant and p-C/EBPα no longer promoted luciferase expression as pGL3-wild type did. Data are averages from three independent experiments; the firefly luciferase activity was normalized to renilla luciferase as a control. *P < 0.05, **P < 0.01.

According to the Ensembl Genome database, the miR-26a gene is located within the intron of the CTD small phosphatase-like protein (CTDSPL) gene. It is likely that the intronic miRNAs are processed from the same primary transcript as the precursor mRNAs, and thus, their expression levels are regulated by the expression of the host mRNA^57^,^58^. To investigate the regulatory pattern of C/EBPα on miR-26a expression in hADSCs, a C/EBPα overexpression plasmid (p-C/EBPα) was constructed and then transfected into hADSCs. Our qPCR results showed that the expression levels of miR-26a and CTDSPL were both greatly promoted by the overexpression of C/EBPα (Fig. 6A,B), suggesting that miR-26a is co-transcribed with CTDSPL. Next, the two 1000-bp fragments within the promoter region of CTDSPL (-2000/-1001 and -1000/-1 from the ATG of CTDSPL) were synthesized and inserted upstream of the firefly luciferase encoding sequence of the pGL3-basic vector. These constructs were termed pGL3-Promoter1 and pGL3-Promoter2, respectively (Fig. 6C). Then, pGL3-Promoter1 and pGL3-Promoter2 were individually co-transfected along with p-C/EBPα into 293T cells. Our luciferase assay showed that the co-transfection of p-C/EBPα and pGL3-Promoter1 significantly promoted luciferase expression compared to the other groups (Fig. 6D). In addition, a chromatin immunoprecipitation (ChIP) assay was performed to determine whether C/EBPα physically binds to the promoter region of CTDSPL. As shown in Fig. 6E, C/EBPα could specifically immunoprecipitate fragments containing Primers A (-1580/-1464, 117-bp), B (-1422/-1301, 122-bp) and C (-1220/1101, 120-bp) within the -2000/-1001 region, and Primer NC (-1786/-1683, 104-bp) was used as a negative control. To further investigate whether these immunoprecipitated fragments had a transcriptionally active response to C/EBPα, three luciferase reporter systems were constructed with the above fragments deleted and referred to as pGL3-ΔA (-1580/-1464, 117-bp deleted), ΔB (-1422/-1301, 122-bp deleted) and ΔC (-1220/1101, 120-bp deleted) in Fig. 6F. Our luciferase assay results showed that the co-transfection of p-C/EBPα and pGL3-ΔA (-1580/-1464, 117-bp deleted) could no longer increase luciferase expression compared to pGL3-ΔB and pGL3-ΔC. According to the transcription factor binding site prediction software Patch 1.0, a putative binding site for C/EBPα (-1530/-1526, GCAAG) was predicted. Luciferase reporter systems containing either the wild-type (-1530/-1526, GCAAG) or mutant (-1530/-1526, ATGGA) binding sites of C/EBPα were constructed and termed pGL3-wild type and pGL3-mutant, respectively (Fig. 6G). Our luciferase assay showed that the mutant binding site had no response to C/EBPα compared with the wild-type site. Taken together, these findings indicated that C/EBPα transcriptionally activates miR-26a expression by directly binding to the CTDSPL promoter region.

**Figure 7:**
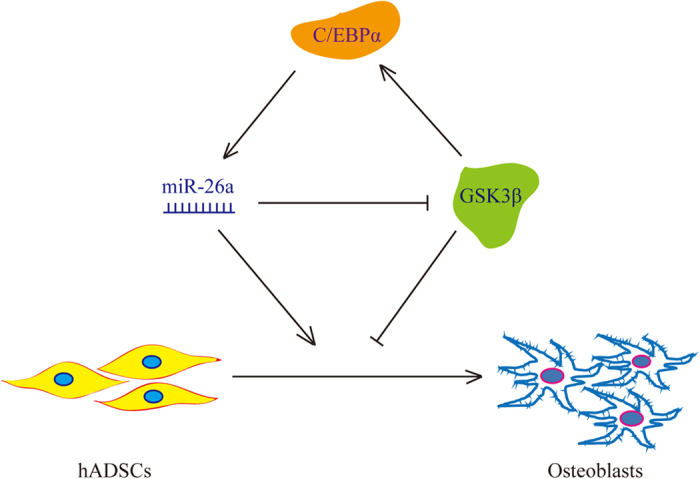
The schematic diagram represents the regulatory loop containing miR-26a, GSK3β and C/EBPα. miR-26a represses GSK3β by directly binding to the 3′UTR of its mRNA. GSK3β affects the intracellular β-catenin content and consequently regulates its downstream target, C/EBPα. C/EBPα reverse transcriptionally regulated miR-26a by physically binding to the CTDSPL promoter region. miR-26a was demonstrated to positively regulate the osteogenesis of hADSCs whereas GSK3β repressed the osteogenic process.

In summary, our data demonstrated that miR-26a is a positive regulator of the osteogenesis of hADSCs. MiR-26a was shown to suppress GSK3β by directly binding to the 3′UTR of its mRNA, and GSK3β was demonstrated to negatively regulate the osteogenesis of hADSCs. GSK3β was also shown to affect the Wnt signalling pathway by regulating β-catenin and subsequently altered the expression of its downstream target C/EBPα. Finally, C/EBPα was demonstrated to transcriptionally regulate the expression of miR-26a by physically binding to the CTDSPL promoter region (Fig. 7).

## Discussion

Human adipose-derived mesenchymal stem cells (hADSCs) have become promising seed cells for bone tissue engineering due to their easy access and availability in large quantities^12^,^59^,^60^. Elucidating the underlying molecular mechanisms that control the osteogenesis of hADSCs will provide deeper insights into the regulatory patterns involved in that process and allow us to develop more efficient methods for curing bone defects. Recently, microRNAs (miRNAs) have emerged as important regulators that affect the osteogenesis of MSCs^61^,^62^. Evidence has indicated that miR-26a is up-regulated in MSCs during osteogenic differentiation, suggesting that miR-26a could be involved in the regulation of osteogenic differentiation^21^,^22^. However, researchers have not reached a consensus about the role of miR-26a in that process^23^,^24^,^25^. In the present study, miR-26a was shown to be up-regulated, in a time-dependent manner, in hADSCs during osteogenic differentiation, suggesting that miR-26a might participate in the osteogenic differentiation process of hADSCs. To elucidate the effects of miR-26a on the regulation of osteogenesis in hADSCs, we performed gain- and loss-of function analyses. The overexpression of miR-26a significantly up-regulated the mRNA and protein expression of osteogenic-specific markers and also increased ALP activity and the promotion of extracellular matrix (ECM) mineralization; the knockdown of miR-26a attenuated these processes. Our data indicated that the overexpression of miR-26a promoted osteogenic differentiation, whereas the knockdown of miR-26a repressed osteogenesis in hADSCs, suggesting that miR-26a acts as a positive regulator of hADSC osteogenic differentiation.

Various studies have demonstrated that miRNAs regulate diverse biological processes by affecting key molecules that control cellular behaviours at a post-transcriptional level^61^,^63^,^64^,^65^. Previous studies have revealed that miR-26a is involved in the regulation of human airway smooth muscle cell hypertrophy and the promotion of hypoxic rat neonatal cardiomyocytes apoptosis through the repression of glycogen synthase kinase 3β (GSK3β)^39^,^40^. GSK3β is one of the key molecules that taking part in diverse molecular pathways, among which Wnt signaling pathway plays important role in regulating the osteogenesis of MSCs^31^. Therefore, we hypothesized that miR-26a might also directly target GSK3β in hADSCs to regulate the osteogenic differentiation process. To test our hypothesis, hADSCs were transduced with miR-26a, miR-26a inhibitor or miR-NC, western blot and qPCR results demonstrated that miR-26a repressed GSK3β protein levels, but did not affect the mRNA expression of GSK3β, indicating that miR-26a repressed GSK3β at a post-transcriptional level. To investigate the regulatory pattern of miR-26a on GSK3β, we identified a putative binding site of miR-26a located at position 4636–4643 of the GSK3β 3′UTR. Luciferase assay revealed that co-transfection of miR-26a and luciferase reporter plasmid containing the wild-type binding site potently repressed luciferase expression levels, suggesting that position 4636–4643 of GSK3β 3′UTR was one of the direct binding sites of miR-26a. Furthermore, to clarify whether the protein repressive effect of miR-26a on GSK3β was mediated by the specific binding of miR-26a to position 4636–4643 of GSK3β 3′UTR and to exclude that secondary structures of the full-length 3′UTR hamper the recognition of the particular binding site, we synthesized the full-length of the 3′UTR of GSK3β carrying either the wild-type or mutant sequence of position 4636–4643. Luciferase assays indicated that the co-transfection of miR-26a and GSK3β 3′UTR-full-wt significantly repressed luciferase expression. Collectively, our data suggested that GSK3β was one of the target genes of miR-26a in hADSCs and that miR-26a repressed GSK3β by specifically binding to position 4636–4643 of the GSK3β 3′UTR.

Regarding the role of GSK3β in osteogenesis, a previous study has demonstrated that GSK3β positively regulates the osteogenesis of murine ADSCs, but other studies have revealed that the inhibition of GSK3β promotes the osteogenic differentiation of MSCs^42^,^43^,^66^,^67^. Thus, our study attempted to determine the regulatory role of GSK3β in hADSC osteogenic differentiation. After transfecting hADSCs with a GSK3β-overexpressing plasmid and siRNA, we found out that the overexpression of GSK3β not only significantly decreased the mRNA and protein expression levels of osteogenic-related markers, but also repressed the ALP activity and the mineralization of extracellular matrix. Besides, GSK3β also negatively regulated the expression of osteogenic-specific transcription factors, such as Ostrix, SATB2 and Runx2, which have been proved to be essential in controlling the osteogenesis^68^,^69^,^70^. Collectively, our data revealed that the overexpression of GSK3β repressed the osteogenic differentiation of hADSCs, whereas the siRNA-mediated knockdown of GSK3β promoted osteogenesis, indicating that GSK3β acts as a negative regulator osteogenesis in hADSCs. Combined with the results that miR-26a directly targets GSK3β by binding to the 3′UTR, we speculated that miR-26a might regulate osteogenic differentiation by inhibiting GSK3β in hADSCs.

GSK3β is a well-known key component of Wnt signaling pathway. The inhibition of GSK3β results in the nucleus aggregation of β-catenin, which forms a complex with the TCF/LEF transcriptional factor family to regulate the expression levels of specific downstream genes^26^,^30^,^31^,^55^. In this study, we performed gain- and loss-of-function analyses using GSK3β overexpressing plasmids and siRNA to investigate the role of GSK3β in regulating β-catenin and downstream target genes. The results of our western blot and cellular immunofluorescence analyses revealed that β-catenin levels were increased by the knockdown of GSK3β and that β-catenin shifted from the cytoplasm to the nucleus under these conditions; in contrast, the overexpression of GSK3β decreased β-catenin levels. Next, we used a luciferase reporter vector containing TCF/LEF responsive elements to detect the activation or repression of the Wnt signalling pathway by GSK3β. Our data showed that the knockdown of GSK3β activated the Wnt signalling pathway and that the overexpression of GSK3β repressed this pathway. Because C/EBPα has been demonstrated to be one of the downstream target genes of the Wnt pathway^43^,^48^,^49^, we used a GSK3β overexpressing plasmid and siRNA to further test whether GSK3β had an influence on the expression of C/EBPα in hADSCs. LiCl and si-β-catenin were used as positive controls in this study because LiCl is able to activate the Wnt signalling pathway, whereas si-β-catenin can repress this pathway^51^. Our qPCR and western blot results indicated that both the knockdown of GSK3β and treatment with LiCl significantly reduced C/EBPα expression, whereas the overexpression of GSK3β and si-β-catenin elevated its expression. Taken together, our data suggested that GSK3β regulated intracellular β-catenin content and localization, subsequently modulates the expression level of its downstream target, C/EBPα.

C/EBPα has been demonstrated to transcriptionally regulate a series of miRNAs^46^,^47^,^71^, and recent studies have revealed that C/EBPα also transcriptionally activate miR-26a in human airway smooth muscle cells by binding to the promoter region of miR-26a^40^. Therefore, we tested whether miR-26a is regulated by C/EBPα in hADSCs and explored the precise regulatory mechanism using luciferase reporter assays and chromatin immunoprecipitation (ChIP). First, according to the Ensembl genome database, miR-26a is located at chromosome 3 and overlaps with CTD small phosphatase-like protein (CTDSPL). Previous research has revealed that intronic miRNAs are produced from the same primary transcript as the precursor mRNAs, and thus, their expression are related to the host mRNA^57^,^58^. To test the regulatory pattern of C/EBPα on miR-26a expression in this study, a C/EBPα expressing vector was transfected into hADSCs and our data showed that miR-26a, along with the expression level of CTDSPL, were dramatically elevated by C/EBPα, suggesting that miR-26a was co-transcribed with CTDSPL. Next, two 1000-bp fragments (-2000/-1001 and -1000/-1 from the ATG of CTDSPL) of the CTDSPL promoter region were cloned into a luciferase reporter vector. Our luciferase assay results indicated that the co-transfection of p-C/EBPα and pGL3-Promoter1 significantly increased luciferase activity, suggesting that the -2000/-1001 fragment had transcriptional response to C/EBPα. Then, chromatin immunoprecipitation (ChIP) was performed, and our results showed that anti-C/EBPα antibody could specifically immunoprecipitate the DNA fragments containing Primer A (-1580/-1464, 117-bp), B (-1422/-1301, 122-bp) and C (-1220/1101, 120-bp), suggesting that C/EBPα might physically bind to the promoter region of miR-26a. Then, three luciferase reporter systems were constructed with the above immunoprecipitated fragments deleted, and our data showed that co-transfection of p-C/EBPα and pGL3-ΔA (-1580/-1464 deletion) could no longer elevate luciferase expression compared with the other combination, suggesting that the 117-bp fragments (-1580/-1464) had a transcriptional response to C/EBPα. Furthermore, the transcription factor binding site prediction software Patch 1.0 showed a putative binding site (GCAAG, -1530/-1526) to which C/EBPα might bind. When the two 1000-bp sequences (-2000/-1001) containing either the wild type (GCAAG, -1530/-1526) or mutant binding site (ATGGA, -1530/-1526) were cloned into luciferase reporter systems, our data revealed that the mutant binding site showed no response to C/EBPα. Above all, our data suggested that C/EBPα transcriptionally activates the expression of miR-26a in hADSCs and that this activation was mediated through the direct binding of C/EBPα to the CTDSPL promoter region.

## Conclusions

Our data demonstrated that the overexpression of miR-26a enhanced hADSC osteogenesis, whereas osteogenesis was repressed by miR-26a knockdown. We further revealed that miR-26a interfered with GSK3β by directly binding to the 3′UTR of its mRNA and that GSK3β served as a negative regulator of osteogenesis in hADSCs. GSK3β was also shown to affect the Wnt signalling pathway through the regulation of β-catenin, which subsequently altered the expression of its downstream target C/EBPα. C/EBPα was found to transcriptionally activate the expression of miR-26a by physically binding to the CTDSPL promoter region. Taken together, our data demonstrated a novel feedback regulatory loop consisting of miR-26a, GSK3β and C/EBPα whose function might contribute to the regulation of hADSC osteogenesis, and our findings will help expand our knowledge about the precise and complex regulatory network controlling cell differentiation.

## Additional Information

**How to cite this article**: Wang, Z. *et al.* A regulatory loop containing miR-26a, GSK3β and C/EBPa regulates the osteogenesis of human adipose-derived mesenchymal stem cells. *Sci. Rep.* **5**, 15280; doi: 10.1038/srep15280 (2015).

## Supplementary Material

Supplementary Information

## Figures and Tables

**Table 1:** Primers used for qPCR.

**Genes**	**Accession No.**	**Forward (5′-3′)**	**Reverse (5′-3′)**	**Annealing temperature (°C)**	**Product size (base pairs)**
Ocn	NM_199173	cactcctcgccctattggc	ccctcctgcttggacacaaag	60	112
BSP	NM_004967	cactggagccaatgcagaaga	tggtggggttgtaggttcaaa	60	106
Runx2	NM_001015051	tggttactgtcatggcgggta	tggttactgtcatggcgggta	60	101
Osx	NM_152860	cctctgcgggactcaacaac	agcccattagtgcttgtaaagg	60	128
OPN	NM_001251830	ctccattgactcgaacgactc	caggtctgcgaaacttcttagat	60	230
GSK3β	NM_001146156	ggcagcatgaaagttagcaga	ggcgaccagttctcctgaatc	60	180
β-catenin	NM_001098209	aaagcggctgttagtcactgg	cgagtcattgcatactgtccat	60	215
C/EBPα	NM_004364	gtggagacgcagcagaag	ttccaaggcacaaggttatc	60	450
GAPDH	NM_001256799	ggagcgagatccctccaaaat	ggctgttgtcatacttctcatgg	60	197

**Table 2:** Primers used for cloning.

**Name**	**Forward (5′-3′)**	**Reverse (5′-3′)**
miR-26a	tgggatccatcctggctgtgctgtgata	ccgctcgagaagcttaaaaaagggcaggagactgatttgtg
p-GSK3β	tccgctcgagatgtcagggcggcccagaac	atggggtaccgtggtggagttggaagctgatg
p-C/EBPα	cgcaaatgggcggtaggcgtg	cgtcgccgtccagctcgaccag
Primer NC	gcctgctggaagccaca	agtgggcggcctgag
Primer A	ctgcggcactaccccg	caaagtgcctcctcagcct
Primer B	tggccagctgccttgc	tgggcattttcgggtgct
Primer C	ctggggccgaatgctgac	gaggggtcccaggagtgag

**Table 3:** Constructed sequences used in this study.

**Name**	**Sequence (5′-3′)**
miR-26a inhibitor	agctaaaaattcaagtaatccaggataggctggatccagcctatcctggattacttgaattttt
GSK3β-3′UTR-wt	aaggactgtgggttgtatacaaactattgcaaacacttgtgcaaatctgtcttgatataaaggaaaagcaaaatctgtataacattattac**tacttgaa**tgcctctgtgactgatttttttttcattttaaatataaacttttttgtgaaaagtatgctcaatgttttttttccctttccccattcccttgtaaataca
GSK3β-3′UTR-mut	aaggactgtgggttgtatacaaactattgcaaacacttgtgcaaatctgtcttgatataaaggaaaagcaaaatctgtataacattattac**acaagttt**tgcctctgtgactgatttttttttcattttaaatataaacttttttgtgaaaagtatgctcaatgttttttttccctttccccattcccttgtaaataca
si-GSK3β-1	Sense: cagcaugaaaguuagcagadtdt Antisense: ucugcuaacuuucaugcugdtdt
si-GSK3β-2	Sense: cauagccgauugcguuaudtdt Antisense: auaacgcaaucggacuaugdtdt
si-GSK3β-3	Sense: cucaagaacugucaaguaadtdt Antisense: uuacuugacaguucuugagdtdt
si-β-catenin-1	Sense: acgacuaguucaguugcuudtdt Antisense: aagcaacugaacuagucgudtdt
si-β-catenin-2	Sense: ccuggugaaaaugcuuggudtdt Antisense: accaagcauuuucaccaggdtdt
si-β-catenin-3	Sense: gugcuaucugucugcucuadtdt Antisense: uagagcagacagauagcacdtdt
