# Supplementary material for: A regulatory loop containing miR-26a, GSK3β and C/EBPα regulates the osteogenesis of human adipose-derived mesenchymal stem cells
Source: Sci Rep. 2015 Oct 15;5:15280. doi: 10.1038/srep15280 (PMC4606799; doi:10.1038/srep15280)
Supplement: Supplementary Information [file srep15280-s1.pdf]

**A regulatory loop containing miR-26a, GSK3β and C/EBPα regulates the osteogenesis of human adipose-derived mesenchymal stem cells**

Zi Wang†, Qing Xie†, Zhang Yu, Huifang Zhou, Yazhuo Huang, Xiaoping Bi, Yefei Wang, Wodong Shi, Hao Sun, Ping Gu* and Xianqun Fan*

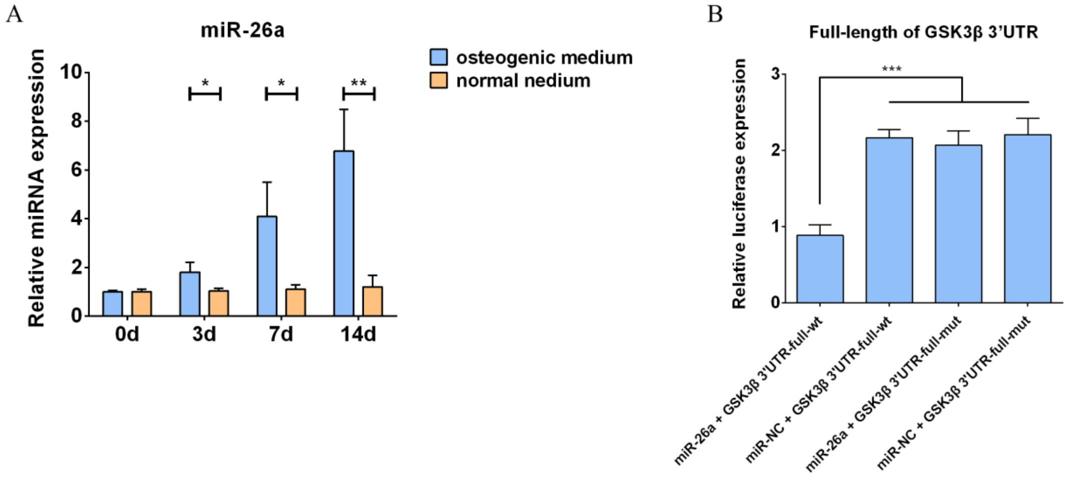


Supplementary Figure S1. (A) qPCR detection of endogenous miR-26a expression levels in hADSCs cultured in osteogenic medium and normal medium, respectively. (B) Dual luciferase reporter assay showed the luciferase expression levels after the co-transfection of miR-26a and the full-length 3'UTR of GSK3β containing either wild-type or mutant binding site. All data were averages from three independent experiments. The firefly luciferase activity data were normalized to renilla luciferase activity. * $P < 0.05$, ** $P < 0.01$, *** $P < 0.001$.
